# Supplementary material for: Non-Redundant Essential Roles of Proteasomal Ubiquitin Receptors Rpn10 and Rpn13 in Germ Cell Formation and Fertility
Source: Cells. 2025 May 12;14(10):696. doi: 10.3390/cells14100696 (PMC12110300; doi:10.3390/cells14100696)
Supplement: Supplementary file 1 [file cells-14-00696-s001.zip › cells-3531184-supplementary.pdf]

Non-redundant essential roles of proteasomal ubiquitin receptors Rpn10 and Rpn13 in germ cell formation and fertility

Supplementary Materials

Table S1. Primer Sequences for Genotyping

Gene	Species	Primer	Sequence
*Rpn13^flox^*	Mouse	Forward primer	5’-GGTGCCTATGTCTCAGCCTCTT-3’
		Reverse primer	5’-GCCATAGAACTGATGAGAGGGAG-3’
*Rpn10^flox^*	Mouse	Forward primer	5’-TGGACCTTCCAGGATGAGGACA-3’
		Reverse primer	5’-GTTCATCTCGGAGCCTGTAGTG-3’
Blimp1-cre	Mouse	Forward primer	5’-CAGACTACCTCAACCGTTCCAC-3’
		Reverse primer	5’-TCCAGCTTTCCCTCCGCATTGA-3’

Table S2. The First and Secondary Antibodies Used in This Study.

Antigen	Label	Host	Clone/Catalogue No	Source	Application
STELLAR	N/A	Rabbit	ab198788	Abcam	IF
SYCP3	N/A	Mouse	ab97672	Abcam	IF; IHC
SYCP3	N/A	Rabbit	ab15093	Abcam	IF
GCNA1[TRA98]	N/A	Rat	ab82527	Abcam	IHC
RPN13	N/A	Rabbit	N/A	Made in our lab.	WB
PLZF	N/A	Mouse	sc28319	Santa Cruz	IHC
DDX4/MVH	N/A	Rabbit	ab13840	Abcam	IHC
SOX9	N/A	Rabbit	ab185966	Abcam	IHC
γH2AX	N/A	Mouse	05-636	Millipore	IF; IHC
SYCP1	N/A	Rabbit	ab15087	Abcam	IF
PSMD2[Rpn1]	N/A	Rabbit	ab197054	Abcam	WB
PSMD4[Rpn10]	N/A	Rabbit	PA5-118210	Thermo Fisher	WB
USP14	N/A	Rabbit	ab192618	Abcam	WB
PSMD14[RPN11]	N/A	Rabbit	4197S	Cell Signaling	WB
UCH37	N/A	Rabbit	ab133508	Abcam	WB
PSME4[PA200]	N/A	Rabbit	ab5620	Abcam	WB
α4s	N/A	Rat	N/A	Made in our lab.	WB
β1	N/A	Mouse	PW8140	Enzo	WB
PSMB4[β7]	N/A	Mouse	sc390878	Santa Cruz	WB
β-actin	N/A	Mouse	A5441	Sigma	WB
Goat Anti-Rat	TRITC	Goat	ZF-0318	ZSGB-BIO	IHC
Goat Anti-Rat	FITC	Goat	ZF-0315	ZSGB-BIO	IHC
Goat Anti-Rabbit	Alexa Fluor	Goat	ZF-0516	ZSGB-BIO	IF; IHC

	594				
Goat Anti-Mouse	Alexa Fluor 594	Goat	ZF-0513	ZSGB-BIO	IF; IHC
Goat Anti-Mouse	FITC	Goat	ZF-0312	ZSGB-BIO	IF; IHC
Goat Anti-Rabbit	FITC	Goat	ZF-0311	ZSGB-BIO	IF; IHC
Goat Anti-Rat	HRP	Goat	ZB-2307	ZSGB-BIO	WB
Goat Anti-Rabbit	HRP	Goat	ZB-5301	ZSGB-BIO	WB
Goat Anti-Mouse	HRP	Goat	ZB-5305	ZSGB-BIO	WB

A

Male(n)	Female(n)	Fertility	Litter numbers	Number of pups	Average litter size
Control	WT	10/11	38	319	8.4
Rpn13-cKO	WT	0/10	0	0	0
WT	Control	13/14	59	364	6.2
WT	*Rpn13-cKO*	0/10	0	0	0

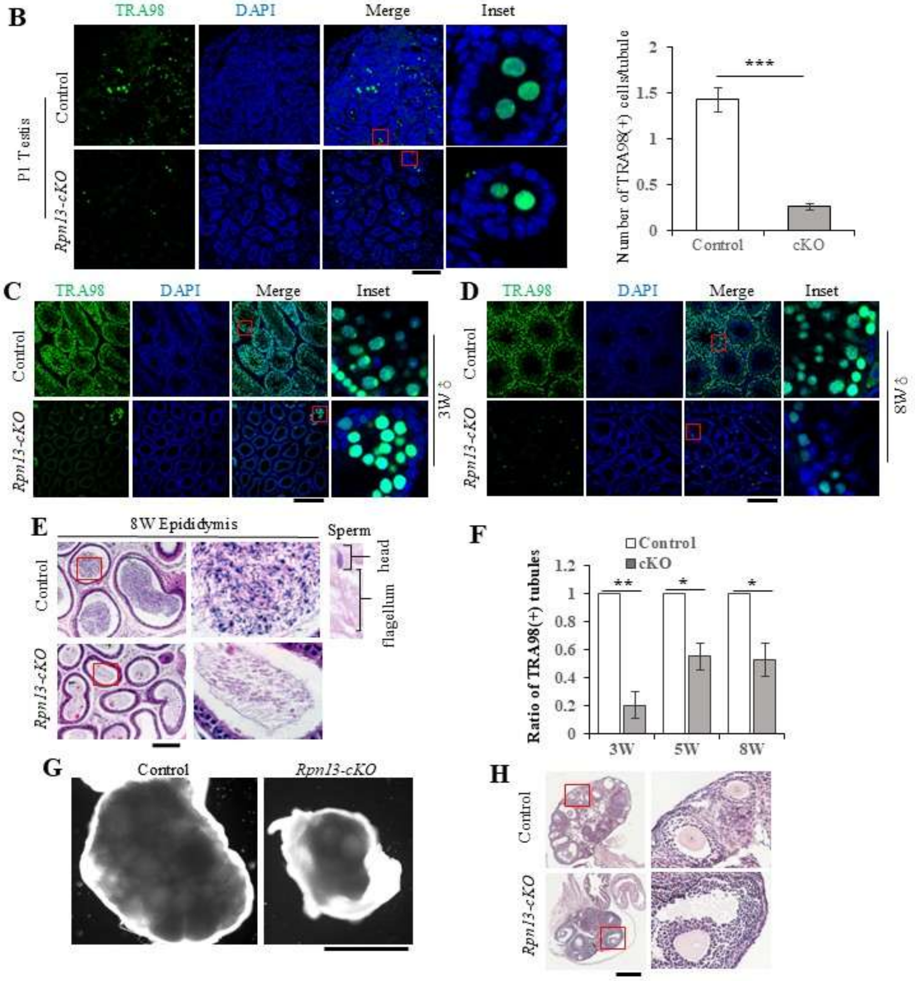

Figure S1. Conditional deletion of Rpn13 leads to infertility in both male and female. (A)The control and *Rpn13-cKO* mice were mated with the WT mice, and numbers of their offsprings were counted. (B) Immunostaining of TRA98 on testicular paraffin sections from the control ($Rpn13^{flox/+}$, $Rpn13^{flox/flox}$ or $Rpn13^{flox/+}$*Blimp1-Cre*) and *Rpn13-cKO* mice at postnatal day 1 The numbers of TRA98-positive cells per tubule were counted. DNA was stained with DAPI. Scale bar, 100 µm. (C-D) Immunostaining of TRA98 from the control ($Rpn13^{flox/+}$ or $Rpn13^{flox/flox}$) and *Rpn13-cKO* testicle at 3 W (C) and 8 W (D) of age. Scale bar, 200 µm. (E) H & E staining of epididymal sections from the control ($Rpn13^{flox/+}$) and *Rpn13-cKO* mice. There had no sperm in *Rpn13-cKO* epididymis. Scale bar, 200 µm. (F) The ratio of TRA98-positive tubules from the control ($Rpn13^{flox/+}$, $Rpn13^{flox/flox}$ or $Rpn13^{flox/+}$*Blimp1-Cre*) and *Rpn13-cKO* testicle at different ages. Two-tailed unpaired

t test, mean with SEM. *P<0.05, **P<0.01.(G)The ovary from the control (*Rpn13*$^{flox/+}$) and *Rpn13-cKO* female mice at 5 W old. Scale bar, 500 μm.(H) H & E staining for ovarian paraffin sections from the control (*Rpn13*$^{flox/+}$) and *Rpn13-cKO* mice at 5 W old. Scale bar, left, 500 μm; right, 100 μm.

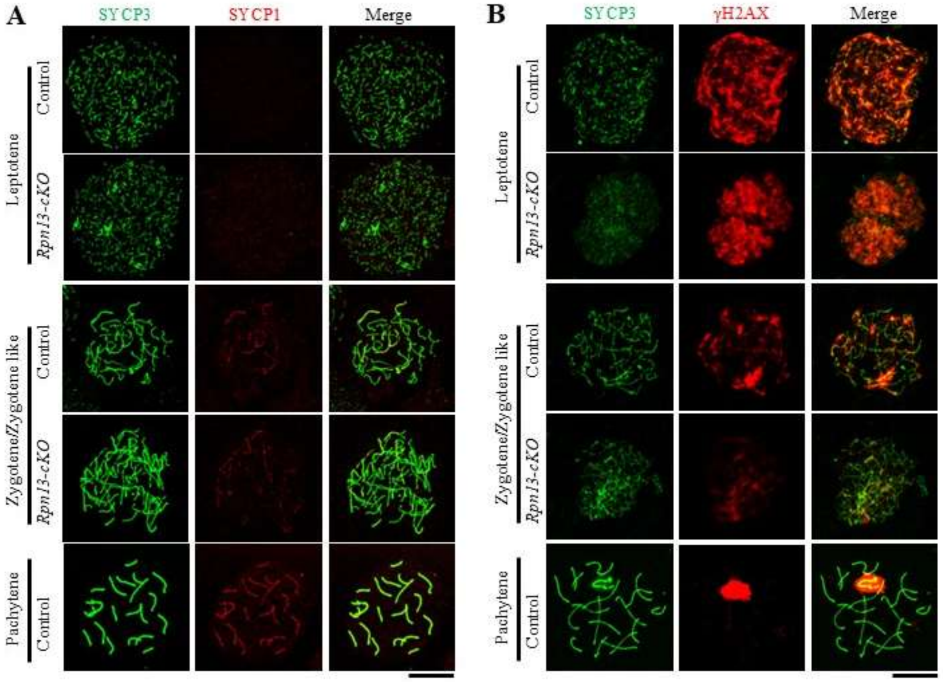

Figure S2. Rpn13 deletion in PGC causes meiotic defect and abnormal spermatogenesis. (A-B) Immunofluorescence staining of SYCP3 and SYCP1(A) or γ-H2AX(B) on nuclear surface spreads of spermatocytes at leptotene stage, zygotene/zygotene-like stage and pachytene stage from the control (*Rpn13*$^{flox/+}$ or *Rpn13*$^{flox/+}$*Blimp1-Cre*) and *Rpn13-cKO* testis at 5 W old. Scale bar, 20 μm.

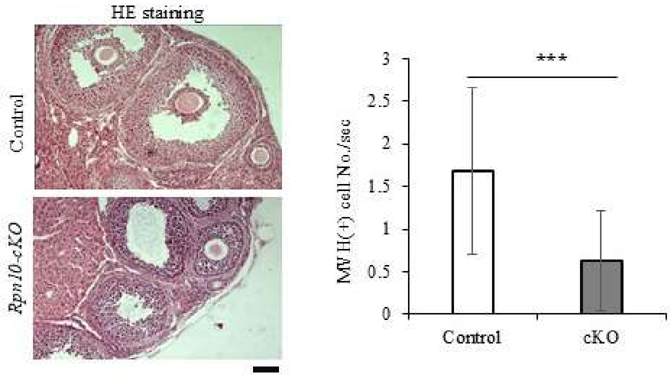

Figure S3. Deletion of Rpn10 in PGCs reduces the number of oocytes. Ovaries from the control and the *Rpn10-cKO* mice at 6 months old were analyzed by H & E staining. Scale bar, 100 μm.
